# Early Functional Recovery Trajectories After Severe Traumatic Brain Injury: A Secondary Analysis of the TBIMS National Database

**DOI:** 10.3390/brainsci16010073

**Published:** 2026-01-06

**Authors:** Andrea Calderone, Lilla Bonanno, Carmela Rifici, Rocco Salvatore Calabrò

**Affiliations:** IRCCS Centro Neurolesi Bonino-Pulejo, S.S. 113 Via Palermo, C.da Casazza, 98124 Messina, Italy; lilla.bonanno@irccsme.it (L.B.); carmela.rifici@irccsme.it (C.R.); roccos.calabro@irccsme.it (R.S.C.)

**Keywords:** severe traumatic brain injury, neurorehabilitation, functional independence measure, Glasgow Outcome Scale—Extended, functional outcome, longitudinal trajectories, TBIMS, prognosis, secondary analysis, registry-based study

## Abstract

**Background:** Survivors of severe traumatic brain injury (TBI) show highly heterogeneous recovery but early functional trajectories across rehabilitation, and their relationship with 1-year outcomes remain poorly characterized. **Methods:** We performed a secondary analysis of the Traumatic Brain Injury Model Systems (TBIMS) National Database. Adults with severe TBI (Glasgow Coma Scale ≤ 8, post-traumatic amnesia (PTA) > 7 days, or neurosurgical intervention) who received inpatient rehabilitation with Functional Independence Measure (FIM) admission and discharge totals and 1-year Glasgow Outcome Scale—Extended (GOS-E) were included (n = 9438). K-means clustering on FIM admission, FIM discharge, and 1-year GOS-E identified early functional recovery trajectories. Multinomial logistic regression related trajectory class to age, sex, PTA duration, rehabilitation length of stay (LOS), and total LOS. Logistic regression examined associations between trajectory class and 1-year home residence and rehospitalization, adjusted for age and sex. **Results:** Three trajectories emerged: low-functioning/limited improvers (28.0%), substantial improvers (33.7%), and high-functioning (38.3%). The low-functioning trajectory showed lower FIM scores at admission and discharge and worse 1-year GOS-E than the other trajectories. In adjusted models, older age, longer PTA, and longer LOS were associated with less favorable trajectories overall, particularly reducing the likelihood of belonging to the high-functioning trajectory. Substantial improvers and high-functioning patients had higher odds of living at home and lower odds of rehospitalization at 1 year than low-functioning patients. **Conclusions:** Simple routine measures can yield interpretable recovery trajectories after severe TBI that may support prognosis, discharge planning, and follow-up; these trajectories should be interpreted as population-level probabilities rather than deterministic predictions for individual patients.

## 1. Introduction

Severe traumatic brain injury (TBI) is a major cause of long-term disability, mortality, and socioeconomic burden worldwide [1,2]. Survivors frequently experience prolonged disorders of consciousness, cognitive and behavioral disturbances, and complex functional limitations that require specialized, intensive neurorehabilitation [3,4,5]. Despite considerable advances in acute neurocritical care and rehabilitation medicine, recovery after severe TBI remains highly heterogeneous, and predicting individual outcomes continues to be challenging [4,5,6,7].

Traditionally, outcome after TBI has been assessed at single time points using global disability scales such as the Glasgow Outcome Scale—Extended (GOS-E) [8]. While these measures are valuable for trials and epidemiological studies, they provide only a static snapshot and do not capture the dynamic nature of recovery [9,10]. In clinical practice, patients and families are often more interested in how recovery will unfold over time, whether improvement will be rapid and early, slow but progressive, or minimal and plateauing, rather than in a single categorical outcome [11].

Over the past decade, several studies in acquired brain injury (ABI), including severe TBI and prolonged disorders of consciousness, have used repeated measures of disability and global outcome to model longitudinal trajectories of recovery [12,13,14,15,16]. In parallel, longitudinal cohorts focusing specifically on patients with prolonged disorders of consciousness have further characterized recovery patterns in this population [17,18,19]. These investigations suggest the existence of distinct recovery phenotypes, such as “rapid improvers”, “slow improvers”, and “non-responders” [14,16,19], and highlight the potential prognostic value of variables such as age, duration of coma or post-traumatic amnesia (PTA), and early functional status [16,20,21]. However, most available studies are single-center or limited to specific national networks, with relatively small sample sizes and restricted generalizability [16,17,18,19]. External validation of recovery trajectories in large, multicenter, longitudinal datasets is lacking, particularly in cohorts receiving comprehensive inpatient neurorehabilitation [14,15,16,19].

The Traumatic Brain Injury Model Systems (TBIMS) National Database is the largest prospective longitudinal cohort of individuals with moderate-to-severe TBI receiving acute care and comprehensive inpatient rehabilitation in specialized centers across the United States [22,23,24]. The TBIMS National Database includes detailed information on demographics, premorbid functioning, injury characteristics, acute care, inpatient rehabilitation (including Functional Independence Measure (FIM) and Disability Rating Scale (DRS) scores at admission and discharge), and long-term outcomes assessed at 1, 2, and 5 years post-injury and every 5 years thereafter [12,13,15,22,23,24]. A de-identified public use data set has been created to support secondary analyses [24,25]. This rich, standardized dataset provides a unique opportunity to characterize early functional recovery trajectories after severe TBI on a large scale [12,13,14,15,22,23,24,25]. In this study, we used the TBIMS National Database Public Use Data Set to model early functional recovery trajectories in adults with severe TBI, from comprehensive inpatient rehabilitation admission through 1-year follow-up. By explicitly treating the TBIMS National Database as a registry of routinely collected clinical data, this study was designed as a hypothesis-driven secondary analysis that leverages the large sample size and standardized assessments while acknowledging the constraints inherent to registry-based research. To our knowledge, recovery trajectories spanning both comprehensive inpatient rehabilitation and 1-year outcomes have not previously been mapped in the TBIMS cohort using a data-driven clustering approach, and external validation of trajectory patterns observed in smaller or single-centre studies remains limited.

Our primary objective was to identify distinct trajectories of functional recovery based on repeated measures of FIM and 1-year GOS-E. Secondary objectives were to describe the demographic, premorbid, injury-related, and early rehabilitation characteristics associated with each trajectory and to examine the relationship between trajectory class, discharge disposition, and rehospitalization within the first post-injury year. We hypothesized that at least three clinically meaningful trajectories would emerge and that younger age, shorter duration of PTA, and better functional status at rehabilitation admission would be associated with more favorable recovery trajectories. Drawing on currently published work, longitudinal studies in severe TBI have either modelled GOS-E trajectories alone, or examined FIM change without integrating global disability into the same latent framework. By jointly modelling FIM total at rehabilitation admission and discharge and 1-year GOS-E, the present analysis aims to provide a multivariate description of early recovery that cannot be obtained from single-scale or single-time-point approaches. In addition, by relating these joint trajectories to subsequent survival, living situation, and rehospitalization, this study could extend prior TBIMS work beyond descriptive courses of global outcome and offer a more clinically actionable picture of how early recovery patterns shape longer-term prognosis.

## 2. Materials and Methods

### 2.1. Study Design, Data Source and Setting

This study was an observational, multicenter, retrospective cohort study based on secondary analysis of de-identified data from the TBIMS National Database Public Use Data Set [22,23,24,25]. The TBIMS National Database is a prospective longitudinal registry of individuals with moderate-to-severe TBI who receive acute hospital care and comprehensive inpatient rehabilitation at TBIMS centers across the United States [22,23,24]. Participants are enrolled during acute hospitalization or inpatient rehabilitation and are followed longitudinally at 1, 2, and 5 years post-injury and every 5 years thereafter. For the present study, we used the TBIMS National Database Public Use Data Set (release November 2025; DOI 10.17605/OSF.IO/A4XZB), which includes de-identified longitudinal data for more than 20,000 individuals with TBI enrolled since 1989 [25]. The dataset used for analysis in this article was obtained under a formal data use agreement from the TBIMS National Data and Statistical Center and was accessed on 2 December 2025. For the present analysis, we retrospectively identified all adults who met the eligibility criteria, received acute hospital care followed by comprehensive inpatient rehabilitation in a TBIMS center, and had a 1-year follow-up assessment available. No new data were collected for this project, and all analyses were conducted exclusively on the de-identified Public Use Data Set prepared by the TBIMS National Data and Statistical Center. There was no direct contact with participants, family members, treating clinicians, or TBIMS centers, and no attempt was made to re-identify individuals. This project therefore represents a secondary analysis of existing registry data, carried out independently of the original TBIMS studies. The design, analysis, and reporting follow current recommendations for observational cohort studies and for investigations based on routinely collected health data (STROBE and the RECORD extension), with particular attention to transparent case selection, explicit operationalization of severe TBI, handling of missing data, and prespecification of primary and secondary outcomes [26,27].

### 2.2. Study Population

**Figure 1:**
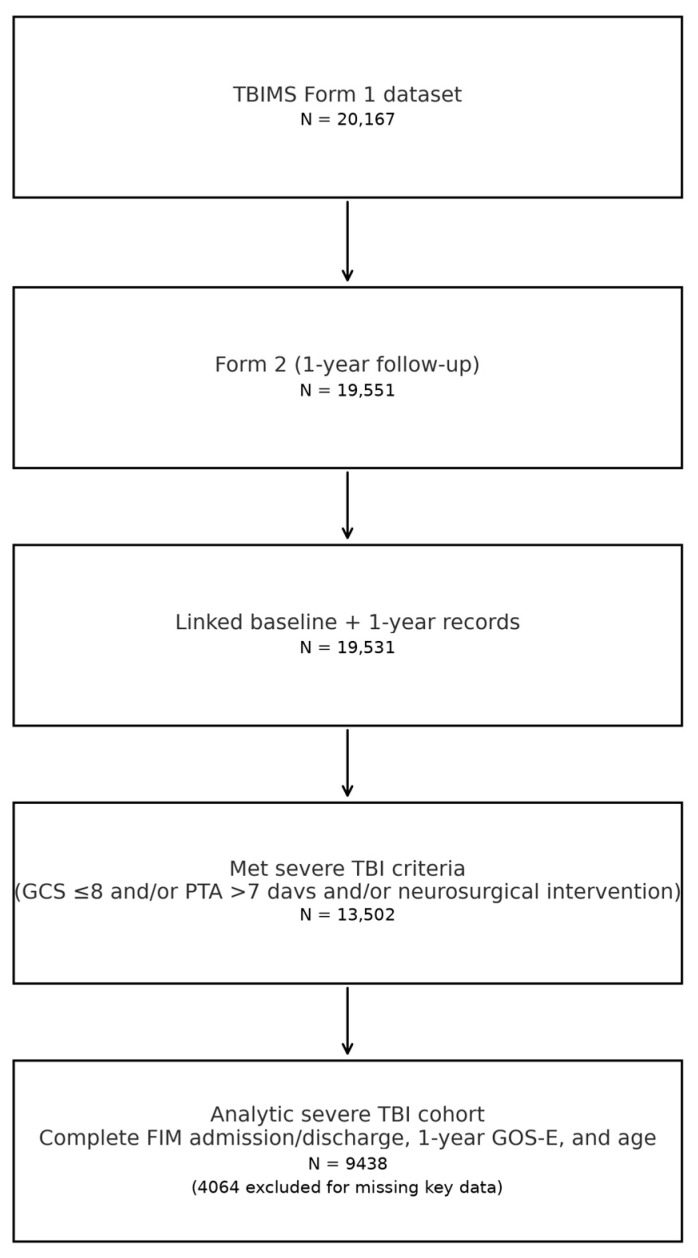
Flow diagram of cohort selection.

The study population was drawn from the prospective TBIMS cohort and consisted of adults with moderate-to-severe TBI who received acute hospital care and comprehensive inpatient rehabilitation in a participating TBIMS center and were enrolled in the National Database. For the present analysis, we restricted the cohort to individuals aged 16 years or older at the time of injury who had completed both the baseline (Form 1) assessment during acute care/rehabilitation and the 1-year follow-up (Form 2). Within this group, we focused on patients with severe TBI, operationalized using variables available in the Public Use Data Set. Specifically, an injury was classified as severe if at least one of the following criteria was met: initial or earliest recorded Glasgow Coma Scale (GCS) total score ≤ 8 after removal of TBIMS-specific missing codes, estimated duration of PTA > 7 days after cleaning of out-of-range values, or neurosurgical intervention for intracranial pathology (craniotomy and/or craniectomy). Where additional markers of severity (such as prolonged loss of consciousness or intracranial pressure monitoring) were available, these were examined descriptively but were not required for inclusion because of variable completeness in the Public Use Data Set. To be eligible for trajectory modelling, participants also had to have valid functional outcome data at the three key time points of interest: FIM total score at rehabilitation admission, FIM total score at rehabilitation discharge, and GOS-E at 1 year post-injury. TBIMS-specific codes indicating that a measure was not assessed or unknown (e.g., 9999 for FIM, 66/99 for GOS-E) were treated as missing and resulted in exclusion from the analytic cohort if they affected any of these three outcomes. We additionally required a valid age at injury; values coded as 777 (≥89 years) were recoded to 89 years in line with TBIMS conventions, whereas values coded as 999 or clearly implausible (e.g., negative values) were treated as missing. Because ages ≥89 years are top-coded in the Public Use Data Set, we could not distinguish recovery patterns among individuals in their 90s and beyond, and we address the potential impact of this recoding in the limitations. Participants with completely missing functional outcome data at all key time points, or with unresolved, clearly implausible, or inconsistent values (such as impossible dates or out-of-range scores that could not be corrected using the TBIMS data dictionary), were therefore excluded. Because the TBIMS National Database is restricted to traumatic brain injuries by design, cases with non-traumatic primary etiology are rare in the Public Use Data Set. Where available variables clearly indicated a non-traumatic cause of the index event, these individuals were excluded to maintain a homogeneous TBI cohort. We did not apply additional systematic exclusions based on pre-existing neurological or neurodegenerative conditions beyond those inherent to the original TBIMS eligibility criteria, as these conditions cannot be reliably identified in a uniform way in the de-identified Public Use Data Set. After applying these criteria and exclusions, the final analytic sample comprised adults with severe TBI who had received acute and inpatient rehabilitation care in TBIMS centers and had complete data on FIM admission, FIM discharge, and 1-year GOS-E, as summarized in the flow diagram (Figure 1). Because the three severity criteria capture overlapping but non-identical aspects of injury severity (depth and duration of impaired consciousness and need for neurosurgical intervention), the resulting cohort includes individuals who met one, two, or all three markers of severe TBI. This heterogeneity should be considered when comparing our findings with cohorts defined using a single criterion, such as admission GCS alone. Injury dates for the analytic cohort ranged from 1992 to 2020, corresponding to the period for which 1-year outcome data and complete functional measures were available in the accessed Public Use Data Set release.

The TBIMS Form 1 dataset initially included 20,167 participants who received acute care and comprehensive inpatient rehabilitation. Among these, 19,531 individuals had matching 1-year follow-up data in Form 2. Severe TBI was operationalised using a combination of GCS ≤ 8, PTA > 7 days, and/or neurosurgical intervention for intracranial pathology, yielding 13,502 severe TBI cases. After excluding 4064 participants with missing or non-interpretable FIM scores at rehabilitation admission or discharge, 1-year GOS-E, or age, the final analytic cohort comprised 9438 individuals with severe TBI. Legend: TBIMS: Traumatic Brain Injury Model Systems; TBI: traumatic brain injury; GCS: Glasgow Coma Scale; PTA: post-traumatic amnesia; FIM: Functional Independence Measure; GOS-E: Glasgow Outcome Scale—Extended.

### 2.3. Outcomes Measures

The primary outcomes of interest were the trajectories of early functional recovery from rehabilitation admission through 1-year post-injury. Functional status during the inpatient rehabilitation episode was assessed using the FIM total score, which ranges from 18 (complete dependence) to 126 (complete independence) and is routinely collected at admission and discharge in TBIMS centers [28,29]. For the purposes of this study, we used the FIM total score at rehabilitation admission and discharge as global indicators of functional independence, without modelling motor and cognitive subscales separately, because the total score was more consistently available in the Public Use Data Set. The one-year global outcome was assessed using the GOS-E, an 8-point scale that classifies disability and recovery from death and vegetative state through severe and moderate disability to lower and upper good recovery [30]. The 1-year GOS-E was treated as an ordered categorical indicator of longer-term functional status and was combined with the FIM admission and discharge scores to construct multivariate profiles of recovery over time. TBIMS-specific codes indicating that FIM or GOS-E scores were not assessed or unknown were treated as missing, and participants with such codes at any of the three key time points were excluded from trajectory modelling. Secondary outcomes were chosen to reflect clinically meaningful aspects of longer-term status and health service utilization. Functional improvement during rehabilitation was summarized descriptively using the change in FIM total score from admission to discharge. At the 1-year follow-up, living situation was used as a proxy for community reintegration and level of independence and was dichotomized as living at home (private residence) versus institutional or other settings. In addition, rehospitalization within the first post-injury year was examined as a marker of medical complexity and ongoing care needs, based on the TBIMS follow-up item asking whether the participant had been rehospitalized since the last assessment. This variable was analyzed as a binary outcome (one or more rehospitalizations versus none) for regression models, while the distribution of the number of rehospitalizations was examined descriptively. Although the TBIMS National Database also includes GOS-E at later follow-up points for some participants, 2-year outcomes were not incorporated into the primary trajectory model in order to focus on early recovery and maximize sample size and completeness of data. Explanatory variables and covariates were drawn from four broad domains: demographic characteristics (including age, sex, race/ethnicity, and education), pre-injury status (such as employment and living situation), injury-related and acute-care variables (including mechanism of injury, initial or best GCS, duration of PTA, neurosurgical procedures, intracranial pressure monitoring, intensive care unit length of stay (LOS), and total acute-care LOS), and rehabilitation-related factors (including time from injury to rehabilitation admission, rehabilitation LOS, and functional status at admission). Where necessary, variables were recoded for clinical interpretability and to ensure adequate cell sizes, for example, by categorizing PTA duration into short, intermediate, and long and expressing LOS variables per 10-day increments. Pre-injury education was categorized into three levels (≤high school, high school diploma/GED/some college, post-secondary/professional) according to the TBIMS EDUCATION codes. Given the large number of potential predictors and the risk of overfitting, a restricted set of clinically relevant covariates, age, sex, PTA duration, rehabilitation length of stay, and total LOS, was selected a priori for inclusion in the primary multinomial models of trajectory class membership, as detailed in Section 2.4. Other variables were used to characterize the cohort and to provide clinical context for the identified trajectories. For the purposes of trajectory modelling, FIM totals and 1-year GOS-E scores were treated as approximately continuous indicators of global functional independence and global outcome, respectively, consistent with common practice in TBIMS analyses and other large TBI cohorts. In line with this assumption, the 1-year GOS-E, although ordinal by design, was handled as an approximately continuous variable for pragmatic reasons so that it could be jointly modelled with FIM totals within the same low-dimensional feature space. In interpreting the results, we focus on broad separation between classes and relative between-class differences rather than on fine-grained interval properties of these scales. We acknowledge that ordinal-aware approaches (e.g., methods that explicitly respect the ordered nature of the GOS-E categories) could preserve distinctions between adjacent outcome levels. However, given the limited number of time points and our primary goal of identifying clinically interpretable broad phenotypes, we adopted this pragmatic approximation and emphasized class separation rather than unit-level score differences.

### 2.4. Statistical Analysis

All analyses were performed on the subset of TBIMS participants who met the operational criteria for severe TBI and had valid functional data at all three key time points (FIM total at rehabilitation admission and discharge and GOS-E at 1 year), as defined in Section 2.2 and Section 2.3. TBIMS-specific codes for missing or non-applicable values were handled according to the official data dictionary. In particular, GCS total scores coded as 77, 88, or 999 were recoded as missing; PTA duration values coded as 8888 or 9999 or taking negative values were treated as missing; FIM total scores coded as 9999 at admission or discharge were considered “not assessed” and set to missing; and ages coded as 999 or negative were treated as missing, while TBIMS code 777 was recoded to 89 years to represent the lower bound of the ≥89 years category. Severe TBI was operationalized using three complementary criteria derived from the Form 1 dataset, namely GCS total ≤ 8 after cleaning, PTA duration > 7 days after removal of extreme codes, or neurosurgical intervention for intracranial pathology (craniotomy and/or craniectomy). Participants meeting at least one of these criteria were classified as having severe TBI.

The analytic cohort for trajectory modeling included all severe TBI participants with non-missing FIM total scores at rehabilitation admission and discharge, a valid 1-year GOS-E score coded between 1 and 8, and a valid age. This yielded a complete-case dataset for the three functional outcomes and age. For secondary analyses that incorporated additional covariates such as PTA duration and length-of-stay measures, we further restricted the sample to individuals with non-missing values for those variables. Given the large sample size and the fact that most missingness reflected non-collection or TBIMS-specific “not assessed/unknown” codes rather than sporadic missing data, we adopted a complete-case approach rather than implementing multiple imputation. This strategy was chosen to maximize internal consistency and transparency, recognizing that it may reduce generalizability if patients with incomplete data differ systematically from those retained. For multivariable models, we further restricted analyses to participants with non-missing data on all predictors included in each model. No statistical imputation was performed, and potential implications of this complete-case strategy are considered in the discussion.

Descriptive statistics were used to summarize demographic, injury-related, and rehabilitation characteristics in the overall analytic cohort and within each trajectory class. Continuous variables are reported as means and standard deviations and, where informative, medians and interquartile ranges; categorical variables are reported as counts and percentages. To identify patterns of early functional recovery, we applied an unsupervised clustering procedure to the three functional outcome variables: FIM total at rehabilitation admission, FIM total at discharge, and 1-year GOS-E. Because these measures have different scales and distributions, all three were standardized (z-scores) prior to clustering. We then used k-means clustering with Euclidean distance to partition individuals into groups with similar profiles across the three time points [31]. This unsupervised approach was conceptualized as a clustering of empirical functional profiles across the three measures, rather than as a formal latent class growth model. Accordingly, the resulting trajectories are best interpreted as data-driven empirical groupings of individuals with similar observed recovery profiles, not as latent growth classes in a strict statistical sense. K-means clustering was implemented using the scikit-learn library (version 1.4) in Python (version 3.11). Candidate models with two, three, and four clusters were fitted and compared using within-cluster sum of squares (inertia) and the clinical interpretability and size of the resulting classes. As additional internal validation criteria, we inspected the average silhouette coefficient and the Calinski-Harabasz index for candidate solutions (k = 2–4), both of which supported the choice of a three-cluster solution. To reduce sensitivity to initial centroid placement, each k-means solution was estimated using multiple random initializations, and we inspected the resulting cluster assignments to confirm that centroids and class sizes were highly similar across runs, indicating good stability of the chosen three-class solution. The three-cluster solution provided a clear improvement in fit over the two-cluster model and yielded clinically distinct and well-sized groups, whereas the four-cluster solution produced an additional small group without a clearly interpretable trajectory pattern. We therefore retained the three-cluster solution as the primary model. Each participant was assigned to the cluster (trajectory class) corresponding to the nearest centroid in the standardized outcome space, and classes were labelled according to their characteristic pattern of functional recovery (low-functioning/limited improvers, substantial improvers, and high-functioning). For clustering, FIM admission, FIM discharge, and 1-year GOS-E scores were standardized to z-scores and combined in a Euclidean distance framework. Although the GOS-E is ordinal by construction, its numeric scores (1–8) were treated as approximately continuous for the purposes of clustering, reflecting a pragmatic choice that facilitates computation of Euclidean distances and is consistent with common practice in large TBI cohorts.

To examine predictors of trajectory class membership, we fitted a multinomial logistic regression model with trajectory class as the dependent variable and the low-functioning trajectory (Class 0) as reference. Based on clinical relevance and prior literature, the model included age (expressed per 10-year increase), sex (female versus male), PTA duration categorized as short (≤7 days, reference), intermediate (8–28 days), or long (>28 days), rehabilitation LOS (per 10-day increase), and total LOS from acute admission to rehabilitation discharge (per 10-day increase) as independent variables. Results are presented as odds ratios (ORs) with 95% confidence intervals (CIs). Multinomial logistic regression and the two binary logistic regression models were estimated with a logit link using maximum likelihood as implemented in the statsmodels package (version 0.14). The potential for collinearity between predictors was assessed by examining correlation matrices and variance inflation factors, and the final model was specified to balance explanatory value and parsimony. Because our primary interest was in patient-level prognostic factors rather than center- or time-specific effects, we did not include TBIMS center or calendar year in these models. Potential implications of this choice, including the possibility of unmeasured center-level and temporal confounding, are discussed in the Section Methodological Considerations and Limitations.

Two additional logistic regression models were used to assess the association between trajectory class and secondary 1-year outcomes. The first examined the odds of living at home (private residence versus institutional/other setting) at 1 year, and the second examined the odds of having at least one rehospitalization in the previous year. In both models, trajectory class was included as a categorical predictor with the low-functioning trajectory as reference, and age and sex were included as covariates. Living situation was derived from the TBIMS follow-up question “Where do you live now?” (ResF), dichotomized as home (private residence) versus institutional/other settings. Rehospitalization was derived from the TBIMS variable REHOSPF (0 = no, 1 = yes), coded as a binary indicator of at least one rehospitalization since the last assessment. This public-use variable does not provide admission diagnoses or reasons for rehospitalization, and therefore, we could not distinguish TBI-related complications from unrelated causes. All statistical analyses were performed in Python (version 3.11). Data cleaning and descriptive statistics were carried out using pandas (version 2.2), unsupervised k-means clustering was implemented with the scikit-learn library (version 1.4), and multinomial as well as binary logistic regression models were fitted using the statsmodels package (version 0.14). Statistical significance was defined as a two-sided *p*-value < 0.05, but in view of the large sample size, emphasis was placed on the magnitude and precision of effect estimates (ORs and 95% CIs) and on their clinical relevance rather than on *p*-values alone.

### 2.5. Ethics

The present study used only de-identified data from the TBIMS National Database Public Use Data Set. The original TBIMS protocols, including enrollment, acute care, rehabilitation, and follow-up procedures, were approved by the Institutional Review Boards of all participating centers, and informed consent was obtained from participants or their legally authorized representatives in accordance with local regulations. The Public Use Data Set is fully de-identified prior to release by the TBIMS National Data and Statistical Center and does not contain direct identifiers or linkage codes that would permit re-identification of individual participants.

Because the study relied exclusively on fully anonymized data obtained from an open-access online database, and no identifiable or sensitive personal information was processed, local ethics committee approval was not required, although all relevant ethical and data-privacy principles were respected. All analyses were conducted in accordance with the principles of the Declaration of Helsinki and applicable regulations governing the secondary use of anonymized health data [32].

## 3. Results

The TBIMS Form 1 dataset included 20,167 unique participants who received acute care and comprehensive inpatient rehabilitation, whereas the 1-year follow-up dataset (Form 2 records with FollowUpPeriod = 1) comprised 19,551 participants with a completed 1-year assessment. Linking Form 1 and the 1-year follow-up data via the common participant identifier yielded 19,531 individuals with both baseline/rehabilitation and 1-year information. Among these, 13,502 participants (69.1%) satisfied at least one of the predefined operational criteria for severe TBI, namely an initial GCS total ≤ 8 after removal of TBIMS missing codes, PTA duration > 7 days after cleaning of extreme codes, or neurosurgical intervention for intracranial pathology. After exclusion of individuals with missing or non-interpretable functional data at one or more key time points (FIM total at rehabilitation admission or discharge coded as 9999, or GOS-E coded as 66 or 99), as well as those with missing age, the final analytic cohort for trajectory modelling comprised 9438 participants with severe TBI, representing 69.9% of the severe TBI group and 48.3% of the merged baseline–follow-up cohort (Figure 1). In this analytic cohort, the mean age at injury was 39.6 years (SD 18.2), with a median of 36 years and an interquartile range from 23 to 52.8 years; approximately one quarter of participants were women (24.9%). Among the 8193 participants with available PTA data, the mean PTA duration was 27.7 days (SD 21.2) and the median 23 days, indicating a substantial period of post-traumatic confusion in most patients. The mean rehabilitation LOS was 27.4 days (SD 25.8; median 21 days), and the mean total LOS from acute admission to rehabilitation discharge was 49.7 days (SD 34.7; median 41 days). Functional status at rehabilitation admission was markedly impaired, with a mean FIM total score of 48.6 (SD 22.4; median 48; IQR 28–66; range 18–125). By rehabilitation discharge, the mean FIM total had increased to 89.7 (SD 22.2; median 93; IQR 80–106; range 18–126), reflecting substantial gains in independence over the inpatient rehabilitation episode. At 1 year after injury, the mean GOS-E was 5.48 (SD 1.90; median 6; IQR 4–7). The distribution of 1-year GOS-E categories showed that 2.8% of participants had died (GOS-E = 1), 0.4% were in a vegetative state (GOS-E = 2), 16.6% had lower severe disability, 14.2% had upper severe disability, 11.2% had lower moderate disability, 22.0% had upper moderate disability, 12.9% had lower good recovery, and 19.9% had upper good recovery. Baseline demographic and premorbid characteristics of the analytic cohort are summarized in Table 1, and key injury- and rehabilitation-related variables are reported in Table 2. Furthermore, to inform potential selection effects, we also compared available baseline demographics and injury characteristics between severe TBI patients included in the analytic cohort and those excluded because of missing age or functional outcome data; these descriptive comparisons are provided in Supplementary Table S1. In brief, patients who were excluded because of incomplete functional data tended to show somewhat more unfavorable profiles, including longer hospital stays and lower early functional scores, compared with those retained in the analytic cohort. Consequently, the exclusion of these cases may have led to a modest underestimation of the most adverse recovery pathways, although the final analytic sample remains large and appears to represent the full spectrum of severe TBI severity observed within the TBIMS program.

**Figure 2:**
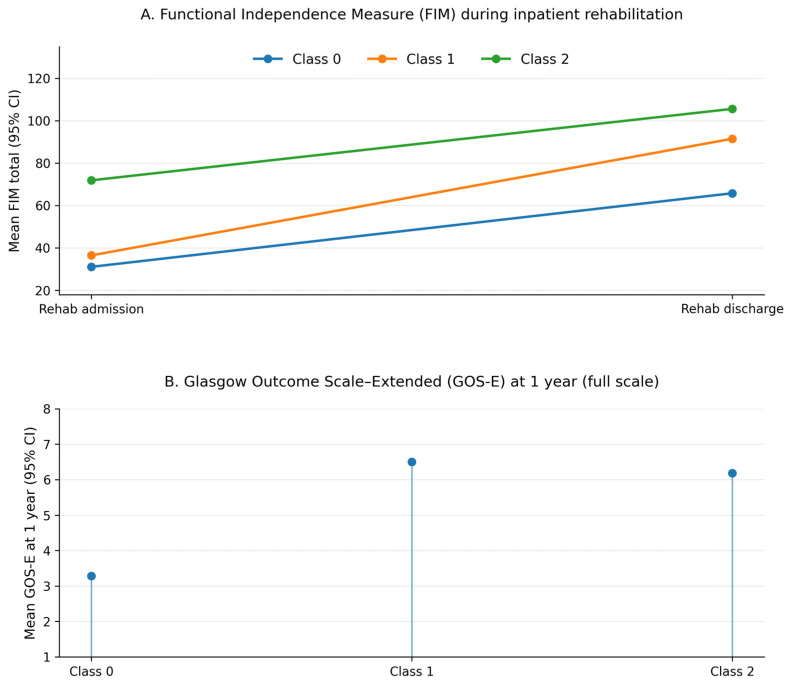
Early functional recovery trajectories after severe traumatic brain injury.

Unsupervised clustering of the standardized FIM admission, FIM discharge, and 1-year GOS-E scores identified three distinct functional recovery trajectories. The three-cluster solution was selected because it provided a better fit than the two-cluster model and yielded classes that were clinically interpretable and adequately sized, whereas a four-cluster solution separated off a small and difficult-to-interpret additional group. In the retained model, the three trajectory classes accounted for 28.0%, 33.7%, and 38.3% of the analytic cohort, respectively. Class 0, comprising 2640 individuals, was characterized by very low functional status at rehabilitation admission and substantial residual disability at both discharge and 1 year. The mean FIM total in this group was 31.1 (SD 13.4) at admission and 65.8 (SD 22.1) at discharge, corresponding to a mean FIM gain of 34.6 points (SD 20.1). At 1 year, the mean GOS-E was 3.28 (SD 1.02), which is consistent with persistent severe disability. Class 1 included 3178 patients who presented with intermediate functional status at admission, achieved large gains during inpatient rehabilitation, and showed relatively favorable 1-year outcomes. In this trajectory, the mean FIM total increased from 36.5 (SD 12.0) at admission to 91.5 (SD 13.0) at discharge, with a mean FIM gain of 55.1 points (SD 17.5), and the mean GOS-E at 1 year was 6.50 (SD 1.20), typically in the upper moderate disability to good recovery range. Class 2 contained 3620 individuals who entered rehabilitation with comparatively high levels of independence and maintained favorable outcomes at follow-up. Their mean FIM total was 71.9 (SD 12.6) at admission and 105.6 (SD 10.3) at discharge, implying a mean gain of 33.7 points (SD 13.3), and the mean 1-year GOS-E was 6.18 (SD 1.55). These functional profiles across time are summarized in Table 3 and illustrated graphically in Figure 2.

Table 4 depicts mean Functional Independence Measure (FIM) total scores at rehabilitation admission and discharge across the three trajectory classes. Table 5 depicts mean 1-year Glasgow Outcome Scale—Extended (GOS-E) scores across classes. Points indicate means, and error bars indicate 95% confidence intervals. Class 0 (low-functioning/limited improvers) shows very low FIM scores at admission, modest gains by discharge, and severe disability at 1 year (mean GOS-E ≈ 3). Class 1 (substantial improvers) starts from intermediate FIM scores, exhibits large FIM gains during rehabilitation, and reaches upper moderate disability to good recovery at 1 year (mean GOS-E ≈ 6.5). Class 2 (high-functioning) enters rehabilitation with relatively high independence, achieves further FIM gains, and maintains favorable 1-year outcomes (mean GOS-E ≈ 6.2).

**Figure 3:**
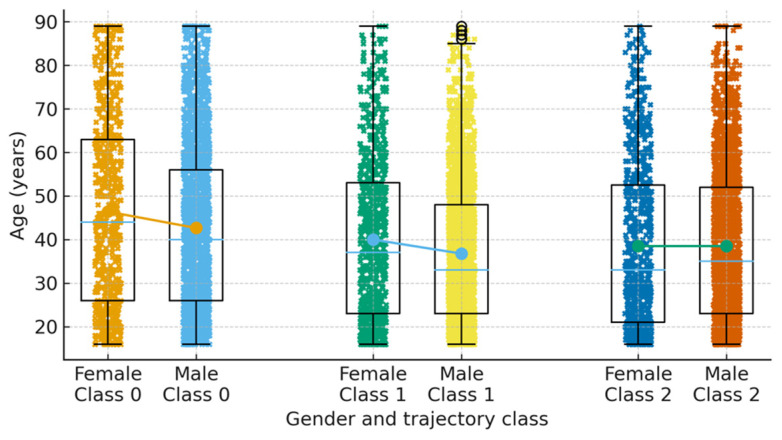
Age distribution by gender across the three recovery trajectory classes.

Baseline and early rehabilitation characteristics differed systematically across the three trajectory classes (Table 2). Patients in the low-functioning trajectory (Class 0) tended to be older (mean 43.6 ± 19.9 years) than those in Class 1 (38.5 ± 17.8 years) and Class 2 (37.5 ± 16.6 years). The proportion of women was similar across classes, ranging from 23.7% in Class 2 to 25.7% in Class 0. PTA duration showed a marked gradient: among participants with available data, the mean PTA duration in Class 0 was 40.5 days (SD 29.6; median 35 days), compared with 30.5 days (SD 17.7; median 28 days) in Class 1 and 19.1 days (SD 13.8; median 16 days) in Class 2. LOS also differed across classes. Individuals in Class 0 had a mean rehabilitation LOS of 41.2 days (SD 36.3; median 30 days) and a mean total LOS of 70.8 days (SD 46.7; median 60 days). In contrast, patients in Class 1 had intermediate LOS values (mean 28.1 ± 20.5 days in rehabilitation and 49.2 ± 25.8 days in total), whereas those in Class 2 had the shortest stays (mean 16.8 ± 12.4 days in rehabilitation and 34.9 ± 20.4 days in total). These patterns suggest that the low-functioning trajectory is associated with older age, more prolonged PTA, and more resource-intensive acute and rehabilitation care, whereas the high-functioning trajectory characterizes younger patients with shorter PTA and shorter hospital stays. The distribution of age by sex within each trajectory class is further illustrated in Figure 3.

Box-and-whisker plots showing age at injury for women and men within each trajectory class (Class 0 = low-functioning, Class 1 = substantial improvers, Class 2 = high-functioning). Individual data points are shown as jittered dots, and large dots connected by lines indicate mean age within each gender–class subgroup.

A multinomial logistic regression model including 8107 participants with complete data on key predictors quantified the independent associations between baseline characteristics and trajectory class membership, as presented in Table 4.

Using the low-functioning trajectory (Class 0) as the reference, increasing age was associated with reduced odds of belonging to either of the more favorable trajectories. For each 10-year increase in age, the odds of being in Class 1 rather than Class 0 decreased by approximately 19% (OR 0.81, 95% CI 0.78–0.84), and the odds of being in Class 2 decreased by approximately 24% (OR 0.76, 95% CI 0.73–0.79). Female sex was not significantly associated with membership in Class 1 compared with Class 0, but women had lower odds of belonging to Class 2 than Class 0 (OR 0.77, 95% CI 0.67–0.90). PTA duration displayed a more complex pattern: compared with short PTA (≤7 days), both intermediate (8–28 days) and long (>28 days) PTA were associated with increased odds of substantial improver status (Class 1 vs. Class 0; OR 1.79, 95% CI 1.33–2.41 and OR 1.68, 95% CI 1.23–2.29, respectively), whereas intermediate and especially long PTA markedly reduced the odds of belonging to the high-functioning trajectory (Class 2 vs. Class 0; OR 0.72, 95% CI 0.55–0.93 and OR 0.20, 95% CI 0.15–0.26, respectively). Longer rehabilitation LOS increased the odds of Class 1 membership (OR 1.09 per 10 additional days, 95% CI 1.03–1.15) but sharply decreased the odds of Class 2 membership (OR 0.57 per 10 additional days, 95% CI 0.53–0.61), consistent with the descriptive observation that high-functioning patients have shorter rehabilitation stays. Total LOS was inversely associated with Class 1 vs. Class 0 (OR 0.79 per 10 days, 95% CI 0.76–0.83), whereas its association with Class 2 vs. Class 0 was modest and did not reach conventional statistical significance.

**Figure 4:**
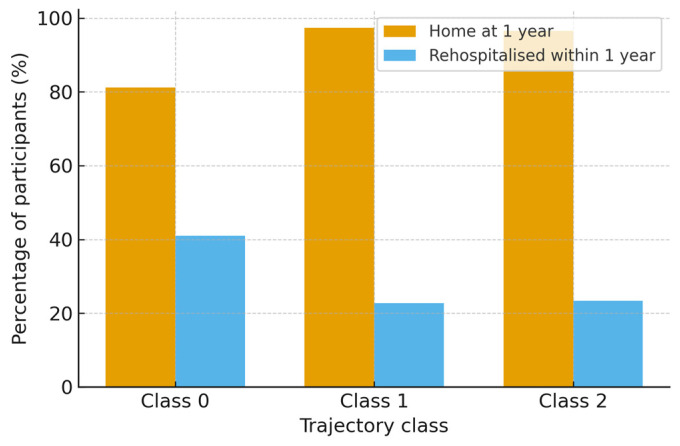
One-year living situation and rehospitalization by recovery trajectory class.

The prognostic relevance of trajectory class was further reflected in 1-year secondary outcomes. Living situation at 1 year was derived from the TBIMS follow-up item on current residence and dichotomized as home (private residence) versus institutional or other setting. Among 9156 participants with valid living situation data, 81.2% of those in Class 0, 97.4% of those in Class 1, and 96.5% of those in Class 2 were living at home. These crude proportions by trajectory class are shown in Figure 4.

Table 4 shows the proportion of participants living at home (private residence) at 1 year after injury, while Table 5 shows the proportion with at least one rehospitalization in the previous year. Compared with the low-functioning trajectory (Class 0), patients in the substantial improver (Class 1) and high-functioning (Class 2) trajectories were much more likely to be living at home (≈97% vs. 81%) and approximately half as likely to have been rehospitalized (≈23% vs. 41%), consistent with the strong association between early recovery profiles and clinically meaningful long-term outcomes.

In a logistic regression model adjusted for age and sex (Table 5), membership in either of the more favorable trajectories was associated with substantially higher odds of home residence at 1 year compared with Class 0. The odds of living at home were more than eightfold higher for participants in Class 1 (OR 8.31, 95% CI 6.52–10.60) and nearly sixfold higher for those in Class 2 (OR 5.83, 95% CI 4.74–7.16). Older age was independently associated with lower odds of home residence, whereas female sex showed a modest positive association.

Rehospitalization within the first post-injury year was also strongly related to trajectory class. Among 9102 participants with valid data, 41.0% of individuals in Class 0 experienced at least one rehospitalization, compared with 22.7% in Class 1 and 23.4% in Class 2. In an age- and sex-adjusted logistic model, both more favorable trajectories were associated with approximately halved odds of rehospitalization relative to the low-functioning trajectory. The ORs were 0.43 (95% CI 0.38–0.49) for Class 1 vs. Class 0 and 0.45 (95% CI 0.40–0.51) for Class 2 vs. Class 0. Increasing age showed a small but statistically significant association with increased risk of rehospitalization, whereas sex was not significantly related to rehospitalization after adjustment. The overall distribution of trajectory class membership and the mean FIM and GOS-E scores by class were stable across alternative reasonable specifications of inclusion criteria, suggesting that the main findings are robust to the pattern of missing data in this dataset.

## 4. Discussion

In this large, multicentre cohort of individuals with severe TBI drawn from the TBIMS National Database, we identified three distinct patterns of early functional recovery spanning the comprehensive inpatient rehabilitation episode and the first year post-injury. Compared with previous TBIMS analyses that have focused on GOS-E courses over time [26] and smaller, single-center studies of functional trajectories, our joint trajectory approach explicitly captures how functional independence (FIM) and global disability (GOS-E) evolve together during the first post-injury year. This combined perspective allows us to distinguish clinical profiles that would appear similar on isolated GOS-E or FIM assessments but diverge in their longitudinal patterns and in their associations with downstream outcomes. Because the TBIMS National Database is a prospective registry of individuals treated in specialized U.S. centers, our findings reflect recovery patterns in survivors who access comprehensive inpatient rehabilitation rather than in all patients with severe TBI, which enhances ecological validity for this population but limits direct generalization to other health systems and care pathways. Roughly one in four patients followed a low-functioning trajectory characterised by very poor functional independence at rehabilitation admission, modest gains during rehabilitation, and a 1-year outcome largely within the severe disability range. About one third of the cohort showed substantial improvement, starting from intermediate functional levels, achieving very large FIM gains during rehabilitation and reaching upper moderate disability to good recovery at 1 year. The remaining group, comprising nearly 40% of the cohort, entered rehabilitation with relatively high functional independence, made further gains and maintained favourable outcomes at 1 year. These trajectories were strongly associated with simple clinical variables available early in the course of care, particularly age, PTA duration and LOS, and with 1-year living situation and rehospitalisation, suggesting that trajectory-based phenotypes capture clinically relevant heterogeneity that is not fully reflected by single time point outcomes.

The present findings are consistent with the growing literature in ABI that describes heterogeneous recovery pathways rather than a single uniform course. Previous single-centre or national cohort studies have identified groups of “rapid improvers”, “slow improvers” and “non-responders”, often using repeated measures of disability or consciousness in specialised neurorehabilitation settings [33,34,35]. Our results complement large contemporary longitudinal cohorts, such as TRACK-TBI and international comparative registries, which have documented wide variability in functional and global outcomes over the first post-injury years despite broadly similar injury severity and advances in acute care [36,37]. Longer-term follow-up studies up to 8–10 years after severe TBI likewise show enduring divergence between favourable and unfavourable courses, with substantial proportions of patients remaining severely or moderately disabled while others return to work and achieve good recovery [38,39,40]. Our analysis extends this work in several ways. First, it leverages a very large, multicentre sample of more than 9 000 individuals with severe TBI drawn from an established national registry, enhancing the generalisability of the observed trajectories. Second, it anchors trajectories across the continuum of care, combining early functional status at rehabilitation admission and discharge with 1-year global outcome, rather than focusing on either the inpatient phase or the follow-up phase in isolation. Third, by relating class membership to 1-year living situation and rehospitalisation, it connects recovery patterns to outcomes that are directly meaningful for patients, families and health systems. Taken together, these results support the concept that severe TBI recovery is best understood as a set of probabilistic pathways, and that simple early indicators can help position patients along these pathways.

The associations between baseline characteristics and trajectory class membership observed in the multinomial models are clinically intuitive but also offer some nuance. The predominance of men in this severe TBI rehabilitation cohort likely reflects the well-established epidemiology of TBI, with higher exposure among males to high-energy mechanisms (e.g., road traffic and occupational injuries) and risk-related activities, which increases the probability of sustaining severe injuries requiring specialized inpatient rehabilitation. Differences in race/ethnicity distribution may similarly reflect underlying population patterns as well as structural and health-system factors (including differential access to trauma and rehabilitation services, referral pathways, and social determinants of health) that shape which patients are captured in the TBIMS program. Accordingly, these sex- and race-related patterns should be interpreted descriptively and may not directly generalize beyond the TBIMS rehabilitation cohort. Similarly, the lower adjusted odds of women belonging to the high-functioning trajectory (Class 2) may reflect a combination of biological and contextual factors (e.g., differences in injury mechanisms, premorbid health/frailty, or care pathways) that are not fully captured in the available TBIMS covariates, and the present analysis cannot disentangle these mechanisms and should be interpreted cautiously.

The low-functioning trajectory was characterised by older age, longer PTA and longer acute and rehabilitation stays, features that align with more severe diffuse injury and medical complexity. This pattern is consistent with prior work showing that PTA duration is one of the most robust predictors of global outcome and functional independence after moderate-to-severe TBI [41,42]. Conversely, the high-functioning trajectory comprised younger patients who emerged more quickly from PTA, entered rehabilitation with relatively high independence and required shorter hospitalisations. The substantial improver trajectory appears to represent an intermediate phenotype: patients with considerable early impairment and relatively long PTA who nonetheless show large functional gains during rehabilitation and good 1-year outcomes. From a clinical standpoint, this group may be particularly important, because it demonstrates that prolonged PTA and longer rehabilitation do not necessarily preclude favourable long-term recovery and may reflect preserved rehabilitation potential rather than futility [38,39,40]. In parallel, participation-focused longitudinal analyses have identified distinct subgroups with delayed but substantial gains in community integration and social roles after TBI, underscoring that early severe limitations do not inevitably translate into poor long-term participation [43].

Trajectory class membership was also strongly linked to 1-year living situation and rehospitalisation, underscoring the real-world implications of early recovery patterns. Patients in the low-functioning trajectory, although still predominantly living at home at 1 year, had a non-trivial proportion in institutional or other non-home settings and almost twice the rehospitalisation rate observed in the more favourable trajectories. In contrast, individuals in the substantial improver and high-functioning trajectories were very likely to be living at home and had substantially lower odds of rehospitalisation. These findings are in line with TBIMS-based and population-based studies reporting substantial and persistent rehospitalisation rates after moderate-to-severe TBI, particularly among individuals with greater disability and medical complexity [44,45,46,47]. Furthermore, rehospitalisation in the first years after injury has been associated with lower participation and social integration at longer-term follow-up, suggesting that it is not only a marker of ongoing medical needs but also of broader vulnerability and societal disadvantage [48]. Taken together, these observations suggest that trajectory-based classifications may help anticipate not only functional outcomes but also service needs and utilization in the post-acute phase and may offer a pragmatic way to operationalize more dynamic, longitudinal prognostic models that go beyond single time-point predictors [49,50]. For example, integrating trajectory information into existing prognostic tools could allow clinicians to identify, early in the course of care, patients who are likely to follow a low-functioning trajectory and thus require proactive planning for long-term support, complex discharge pathways, and closer outpatient monitoring, while at the same time recognizing substantial improvers and reinforcing the value of intensive rehabilitation despite severe initial presentations.

An important aspect of this study is the use of relatively simple measures, FIM totals at rehabilitation admission and discharge and 1-year GOS-E, to construct recovery trajectories. Although more granular domain-specific measures (e.g., detailed cognitive tests, participation scales) would provide additional insights, FIM and GOS-E are widely used, readily interpretable and routinely collected in many rehabilitation systems. Prior trajectory analyses based on FIM and related functional measures have shown that even coarse global indices can capture clinically meaningful patterns of change over time [33,34,35,51,52]. The fact that three clinically meaningful trajectories emerge from these basic metrics suggests that trajectory-based thinking can be implemented using data that are feasible to obtain in routine practice. Moreover, the strong associations between trajectories and PTA duration, a standard indicator of severity that is familiar to clinicians, offers a potential bridge between traditional severity classifications and longitudinal prognosis.

### Methodological Considerations and Limitations

Several methodological considerations and limitations should temper interpretation. First, this is a secondary analysis of registry data, and we were constrained by the variables and coding schemes available in the TBIMS Public Use Data Set. Severity was operationalised using a combination of GCS, PTA duration and neurosurgical intervention, which may misclassify some individuals at the margins of the severe category, particularly when key variables were missing or coded as “unknown”. We adopted a conservative approach to TBIMS-specific codes by recoding non-assessed or unknown values as missing, and we restricted our analytic cohort to participants with valid functional data at all three time points. This complete-case strategy ensures internal consistency but inevitably excludes individuals with incomplete assessments, who may differ systematically from those retained. The fact that approximately 30% of severe TBI cases were excluded from trajectory modelling because of missing functional or age data raises the possibility of selection bias, although the distribution of severity indicators and lengths of stay in the analytic cohort remained broadly consistent with the underlying severe TBI population. Together, these observations suggest that, although selection bias cannot be excluded, the identified trajectories are unlikely to be driven solely by patterns of missing data. Nevertheless, we cannot exclude the possibility that unmeasured factors related to missingness, such as social disadvantage, geographic barriers, or early mortality outside participating centers, may have led us to underestimate the prevalence of the most unfavorable trajectories or to overestimate the proportion of patients achieving good recovery.

Second, we used k-means clustering on standardised functional outcomes rather than a full latent class growth modelling framework. While k-means is a simple and transparent method for grouping individuals based on multivariate profiles, it does not explicitly model measurement error, does not allow for flexible non-linear trajectories over more than a few time points and assumes spherical clusters in the feature space. Our choice was motivated by the availability of three key time points, the scale of the dataset and practical constraints of the analytic environment. The resulting trajectories should therefore be interpreted as empirical groupings of patients with similar functional profiles at admission, discharge and 1 year, rather than as latent growth classes in a strict statistical sense. Nonetheless, the three clusters are clinically plausible, show clear gradients in independent variables and are strongly associated with external outcomes, which supports their construct validity. Future work using more complex longitudinal models on the full TBIMS dataset could refine these trajectory patterns and test the stability of the present findings. In addition, treating the ordinal GOS-E scores as approximately continuous when constructing the clustering features may obscure finer distinctions between adjacent outcome categories, and ordinal-aware clustering or latent class approaches may be considered in future work to assess the sensitivity of class definitions to this modelling choice.

Third, we did not include the TBIMS center or calendar year as covariates in our regression models. Rehabilitation practices, resource availability, and discharge policies may have evolved over the nearly three decades covered by the database and may vary across centers. As a result, unmeasured center-level and temporal factors could contribute to the observed associations between patient-level predictors, trajectories, and 1-year outcomes. Accordingly, these estimates should be interpreted as average associations across centers and eras rather than center-specific effects. Future work using multilevel or cross-classified models could more explicitly quantify the extent to which trajectory membership and downstream outcomes differ by center and era of care.

Fourth, although the TBIMS National Database is a major strength in terms of size, prospective design and standardised assessments, it is limited to individuals treated in specialised TBI centres in the United States. As such, our findings may not generalise to patients managed in settings with different acute care resources, rehabilitation intensity or community support structures, or to non-traumatic acquired brain injuries. In addition, we focused on a relatively small set of predictors, age, sex, PTA duration and LOS, that capture broad aspects of injury severity and resource use but do not encompass the full spectrum of factors that may shape recovery. We deliberately did not include FIM admission scores as independent predictors in the primary regression models, because they are themselves used to define the trajectory classes and would therefore introduce circularity into the analysis. Furthermore, we did not include neuroimaging markers, detailed cognitive or behavioural measures, comorbidities, socioeconomic variables or post-acute interventions in the present analysis, although such factors may be important determinants of trajectory membership and could be incorporated in future models. This is particularly relevant in light of evidence that structural MRI findings and blood biomarkers can improve prognostic accuracy beyond clinical variables alone and may help refine risk stratification and pathway assignment in TBI [49,50].

Fifth, our secondary outcomes, living at home and rehospitalisation at 1 year, are derived from single items and dichotomised for analytic simplicity. Living situation does not distinguish between independent living and substantial dependence on caregivers within the home, nor does it capture quality of life or participation. Rehospitalisation is recorded as a yes/no indicator and does not differentiate between elective procedures and emergency admissions or between TBI-related and unrelated causes. These simplifications are inherent in the available data, and they mean that the associations reported here should be interpreted as broad signals rather than precise estimates of specific health system outcomes. However, they do capture domains that are highly salient to patients and families and align with health system priorities, which supports their use as pragmatic indicators of real-world impact [44,45,46,47,48]. Nevertheless, the magnitude and consistency of the associations support the notion that early functional trajectories have meaningful implications for post-acute service use and living arrangements. Sixth, age is top-coded in the TBIMS Public Use Data Set, and participants aged ≥89 years are coded as 777 and were recoded to 89 years. This convention may attenuate heterogeneity among the oldest-old and could modestly affect trajectory assignments in this subgroup; future work using non-top-coded age data, or targeted sensitivity analyses within the oldest age strata, is warranted.

Despite these limitations, this study has several notable strengths. It is, to our knowledge, one of the largest analyses to date to characterise early functional recovery trajectories after severe TBI using a national longitudinal registry. The use of standardised, widely adopted outcome measures across multiple centres enhances both internal validity and external applicability. The trajectory approach explicitly incorporates the dynamic nature of recovery, moving beyond single end-point outcomes to more nuanced patterns that align with clinical observation [33,34,35,51,52]. By linking these trajectories to simple early indicators and to 1-year living situation and rehospitalisation, the study suggests a coherent framework that bridges severity assessment, rehabilitation planning and longer-term prognostic counselling. A further strength is that the joint trajectory classes offer a compact and empirically derived way to communicate prognosis that is closer to the clinical course experienced by patients and families than isolated snapshots of outcome at single time points. In this sense, the present work complements existing TBIMS prognostic tools by providing a trajectory-based framework that could be adapted for early risk stratification and follow-up stratagems in routine care.

Future research could extend this work in several directions. One priority is to integrate more detailed clinical and neurobiological information into trajectory models, including structural and functional neuroimaging, neurophysiological markers and domain-specific cognitive and behavioural assessments, in order to refine trajectory phenotypes and explore underlying mechanisms [49,50]. Another is to examine whether trajectory-based prognostic tools improve communication with families and support decision-making about the intensity and duration of rehabilitation in prospective clinical studies. It will also be important to investigate the stability of trajectories over longer follow-up periods beyond 1 year and to determine how late changes in function or participation relate to early trajectory membership [38,39,40,51,52]. Finally, replicating and adapting this approach in other health systems and in non-traumatic ABI populations could help to establish a more general taxonomy of recovery pathways in severe brain injury.

## 5. Conclusions

In this large, multicenter cohort of individuals with severe TBI, we identified three distinct early functional recovery trajectories spanning comprehensive inpatient rehabilitation and the first year post-injury. Approximately one quarter of patients followed a persistently low-functioning pathway, one third showed substantial improvement from marked dependence to relatively good 1-year outcomes, and nearly 40% were high-functioning from early rehabilitation onward. These trajectories were strongly associated with simple early clinical variables, particularly age, PTA duration, and LOS, and were closely linked to 1-year living situation and rehospitalization risk.

Our findings support a shift from static, single-time-point prognostication towards a more trajectory-based view of recovery in severe TBI. Using routine measures such as FIM and GOS-E, clinicians may be able to position patients along probabilistic recovery pathways that are both clinically interpretable and relevant for families and service planning. Future work should refine these phenotypes with richer clinical and neurobiological data and test whether trajectory-informed prognostic tools improve communication, rehabilitation decision-making, and long-term outcomes in severe TBI. Before being adopted in routine practice, trajectory-based tools derived from the TBIMS cohort should be externally validated in other health systems and care pathways, and their incremental value over existing prognostic models should be demonstrated in prospective studies.

## Figures and Tables

**Table 1:** Demographic characteristics of the severe TBI analytic cohort (N = 9438).

Characteristic	Category	N with Data	Value
Age at injury, years	Overall	9438	39.6 ± 18.2
Sex	Male	9438	7088 (75.1%)
Sex	Female	9438	2347 (24.9%)
Sex	Unknown	9438	3 (0.0%)
Race/ethnicity ^1^	White	9438	6393 (67.7%)
Race/ethnicity ^1^	Black	9438	1604 (17.0%)
Race/ethnicity ^1^	Hispanic	9438	1048 (11.1%)
Race/ethnicity ^1^	Asian	9438	226 (2.4%)
Race/ethnicity ^1^	Native American	9438	58 (0.6%)
Race/ethnicity ^1^	Other	9438	108 (1.1%)
Race/ethnicity ^1^	Unknown	9438	1 (0.0%)
Education level (pre-injury) ^2^	≤High school	9432	2165 (22.9%)
Education level (pre-injury) ^2^	High school diploma/GED/some college	9432	3304 (35.0%)
Education level (pre-injury) ^2^	Post-secondary/professional	9432	3890 (41.2%)
Education level (pre-injury) ^2^	Unknown	9432	79 (0.8%)
Pre-injury employment status ^3^	Employed/working or student	9438	8750 (92.7%)
Pre-injury employment status ^3^	Not employed	9438	648 (6.9%)
Pre-injury employment status ^3^	Unknown	9438	40 (0.4%)

^1^ Race categories derived from TBIMS Race variable. ^2^ Education categories were derived from the TBIMS EDUCATION variable and grouped into three levels: ≤high school, high school diploma/GED/some college, and post-secondary/professional; code 999 was treated as unknown. ^3^ Employment categories derived from TBIMS EMPLOYMENT codes and grouped into “employed/working or student”, “not employed”, and “unknown”; 999 coded as unknown. Legend: General Educational Development (GED).

**Table 2:** Baseline demographic, injury and rehabilitation characteristics by trajectory class (severe TBI analytic cohort, N = 9438).

Characteristic	Overall	Class 0	Class 1	Class 2
Age, years, mean ± SD	39.6 ± 18.2	43.6 ± 19.9	38.5 ± 17.8	37.5 ± 16.6
Sex, n (%)	Male 7088 (75.1)	Male 1960 (74.3)	Male 2365 (74.5)	Male 2763 (76.3)
	Female 2347 (24.9)	Female 679 (25.7)	Female 811 (25.5)	Female 857 (23.7)
	Unknown 3 (0.0)	Unknown 1 (0.0)	Unknown 2 (0.1)	Unknown 0 (0.0)
PTA duration, days, mean ± SD ^1^	27.7 ± 21.2	40.5 ± 29.6	30.5 ± 17.7	19.1 ± 13.8
PTA duration, median (IQR), days ^1^	23 (14–36)	35 (20–53)	28 (18–38)	16 (10–25)
Rehabilitation LOS, days, mean ± SD	27.4 ± 25.8	41.2 ± 36.3	28.1 ± 20.5	16.8 ± 12.4
Rehabilitation LOS, median (IQR), days	21 (13–33)	30 (17–51)	23 (15–35)	14 (10–21)
Total LOS (acute + rehab), mean ± SD ^2^	49.7 ± 34.7	70.8 ± 46.7	49.2 ± 25.8	34.9 ± 20.4
Total LOS, median (IQR), days ^2^	41 (28–61)	60 (40–86)	44 (32–60)	30 (23–42)

^1^ PTA duration available for 8193 participants (Class 0: n = 1739; Class 1: n = 2938; Class 2: n = 3516). ^2^ Total length of stay available for 9328 participants (Class 0: n = 2601; Class 1: n = 3142; Class 2: n = 3585).

**Table 3:** Functional status at rehabilitation admission, discharge, and 1-year follow-up by trajectory class.

Outcome Measure	Overall (N = 9438)	Class 0 (N = 2640)	Class 1 (N = 3178)	Class 2 (N = 3620)
FIM total at rehab admission, mean ± SD	48.6 ± 22.4	31.1 ± 13.4	36.5 ± 12.0	71.9 ± 12.6
FIM total at rehab discharge, mean ± SD	89.7 ± 22.2	65.8 ± 22.1	91.5 ± 13.0	105.6 ± 10.3
ΔFIM (discharge–admission), mean ± SD	41.2 ± 19.6	34.6 ± 20.1	55.1 ± 17.5	33.7 ± 13.3
GOS-E at 1 year, mean ± SD	5.48 ± 1.90	3.28 ± 1.02	6.50 ± 1.20	6.18 ± 1.55

FIM = Functional Independence Measure; GOS-E = Glasgow Outcome Scale—Extended (range 1–8).

**Table 4:** Predictors of trajectory class membership and 1-year outcomes. Multinomial logistic regression for trajectory class membership (reference = Class 0).

Predictor (Per Unit or Category)	Substantial Improver vs. Low-Functioning/Limited Improver OR (95% CI)	High-Functioning vs. Low-Functioning/Limited Improver OR (95% CI)
Age (per 10-year increase)	0.81 (0.78–0.84)	0.76 (0.73–0.79)
Female sex (vs. male)	0.91 (0.79–1.04)	0.77 (0.67–0.90)
PTA 8–28 days (vs. ≤7 days)	1.79 (1.33–2.41)	0.72 (0.55–0.93)
PTA > 28 days (vs. ≤7 days)	1.68 (1.23–2.29)	0.20 (0.15–0.26)
Rehabilitation LOS (per 10-day increase)	1.09 (1.03–1.15)	0.57 (0.53–0.61)
Total LOS (per 10-day increase)	0.79 (0.76–0.83)	0.96 (0.91–1.00)

Model based on N = 8107 participants with complete data on all predictors. Low-functioning/limited improver = trajectory with lowest functional status at admission, discharge and 1 year; substantial improver = intermediate trajectory with large rehabilitation gains; high-functioning = trajectory with highest functional status and shortest stays.

**Table 5:** Predictors of trajectory class membership and 1-year outcomes. Association between trajectory class and 1-year outcomes (logistic regression, adjusted for age and sex; reference = Class 0).

Outcome and Predictor	OR (95% CI)
Living at home at 1 year (vs. institutional/other)	
– Substantial improver vs. low-functioning/limited improver	8.31 (6.52–10.60)
– High-functioning vs. low-functioning/limited improver	5.83 (4.74–7.16)
– Age, per 1-year increase	0.98 (0.98–0.98)
– Female sex (vs. male)	1.24 (1.02–1.51)
Rehospitalisation within 1 year (yes vs. no)	
– Substantial improver vs. low-functioning/limited improver	0.43 (0.38–0.49)
– High-functioning vs. low-functioning/limited improver	0.45 (0.40–0.51)
– Age, per 1-year increase	1.01 (1.00–1.01)
– Female sex (vs. male)	1.04 (0.94–1.16)

## Data Availability

The data that support the findings of this study are available from the Traumatic Brain Injury Model Systems (TBIMS) National Data and Statistical Center (NDSC). De-identified data (TBIMS National Database Public Use Data Set; released November 2025; https://doi.org/10.17605/OSF.IO/A4XZB) can be accessed by qualified investigators upon completion of a Data Use Agreement through the TBIMS NDSC. The authors are not permitted to redistribute the dataset directly.

## Supplementary Materials

The following supporting information can be downloaded at https://www.mdpi.com/article/10.3390/brainsci16010073/s1: Supplementary Table S1: Baseline demographic and injury characteristics in severe TBI patients included in the analytic cohort versus those excluded because of missing age or functional outcome data.
