# Supplementary material for: Early Functional Recovery Trajectories After Severe Traumatic Brain Injury: A Secondary Analysis of the TBIMS National Database
Source: Brain Sci. 2026 Jan 6;16(1):73. doi: 10.3390/brainsci16010073 (PMC12838949; doi:10.3390/brainsci16010073)
Supplement: Supplementary file 1 [file brainsci-16-00073-s001.zip › brainsci-4069971-supplementary.pdf]

Supplementary Table S1. Baseline demographic and injury characteristics in severe TBI patients included in the analytic cohort versus those excluded because of missing age or functional outcome data.

Characteristic	Included severe TBI (analytic cohort, N = 9.438)	Excluded severe TBI (missing age and/or functional outcome, N = 4.444)
Age at injury, years	Mean ± SD: 39.6 ± 18.2 (N = 9 438)	Mean ± SD: 39.1 ± 17.9 (N = 4 422)
Sex, n (%)	Male 7.088 (75.1%)<br>Female 2.347 (24.9%)<br>Unknown 3 (0.0%)	Male 3.441 (77.4%)<br>Female 1.001 (22.5%)<br>Unknown 2 (0.0%)
Race/ethnicity, n (%)	White 6.393 (67.7%)<br>Black 1.604 (17.0%)<br>Hispanic 1.048 (11.1%)<br>Asian 226 (2.4%)<br>Native American 58 (0.6%)<br>Other 108 (1.1%)<br>Unknown 1 (0.0%)	White 2.365 (53.2%)<br>Black 930 (20.9%)<br>Hispanic 599 (13.5%)<br>Asian 139 (3.1%)<br>Native American 24 (0.5%)<br>Other 62 (1.4%)<br>Unknown 325 (7.3%)
Cause of injury, n (%)	Motor vehicle / traffic 4.974 (52.7%)<br>Fall 2.389 (25.3%)<br>Violence 1.023 (10.8%)<br>Other / unknown 1.052 (11.1%)	Motor vehicle / traffic 2 184 (49.1%)<br>Fall 1.050 (23.6%)<br>Violence 661 (14.9%)<br>Other / unknown 549 (12.4%)
Initial GCS total, n (%)	3–8 / 2.204 (23.4%)<br>9–12 / 846 (9.0%)<br>13–15 / 2.144 (22.7%)<br>Unknown / not recorded 4 244 (45.0%)	3–8 / 1.190 (26.8%)<br>9–12 / 457 (10.3%)<br>13–15 / 973 (21.9%)<br>Unknown / not recorded 1 824 (41.0%)
Post-traumatic amnesia duration, n (%)	≤7 days 664 (7.0%)<br>8–28 days 4.394 (46.6%)<br>>28 days 3.135 (33.2%)<br>Unknown / still in PTA / not recorded 1.245 (13.2%)	≤7 days 314 (7.1%)<br>8–28 days 1 970 (44.3%)<br>>28 days 1 544 (34.7%)<br>Unknown / still in PTA / not recorded 616 (13.9%)
Craniotomy, n (%)	Yes 3.085 (32.7%)<br>No 5.306 (56.2%)<br>Unknown 1.047 (11.1%)	Yes 1.265 (28.5%)<br>No 1.809 (40.7%)<br>Unknown 1.370 (30.8%)

Severe TBI was defined as meeting at least one of the following criteria: (i) Glasgow Coma Scale total score 3–8 after resuscitation, excluding codes for chemically paralyzed, intubated, or unknown; (ii) post-traumatic amnesia > 7 days, excluding special missing codes; or (iii) emergency neurosurgical craniotomy or craniectomy. The "excluded" group comprises severe TBI patients who did not meet inclusion criteria for the analytic cohort because of missing age and/or incomplete Functional Independence Measure (admission and discharge) and/or 1-year GOS-E data.
